# P-2338. Global surveillance of human Metapneumovirus (hMPV) and Respiratory Syncytial Virus (RSV) epidemiology since 2022

**DOI:** 10.1093/ofid/ofae631.2490

**Published:** 2025-01-29

**Authors:** Marie-Noelle Billard, Joanne G Wildenbeest, Olivier Braas, Rob Schneijdenberg, Barry Rodgers-Gray, John Fullarton, Louis J Bont

**Affiliations:** University Medical Centre Utrecht, Utrecht, Utrecht, Netherlands; University Medical Centre Utrecht, Utrecht, Utrecht, Netherlands; University Medical Centre Utrecht, Utrecht, Utrecht, Netherlands; University Medical Centre Utrecht, Utrecht, Utrecht, Netherlands; Violicom Medical Limited, Aldermaston, England, United Kingdom; Violicom Medical Limited, Aldermaston, England, United Kingdom; University Medical Centre Utrecht, Utrecht, Utrecht, Netherlands

## Abstract

**Background:**

Human metapneumovirus (hMPV) and respiratory syncytial virus (RSV) are common respiratory pathogens. Whilst the seasonality of RSV is well-documented, less is known about the epidemiology of hMPV and the global impact of the SARS-CoV-2 pandemic on both viruses. The objective of this study was to monitor the real-time temporal and geographic distribution patterns of hMPV and RSV over two seasons.

**Methods:**

A global surveillance dashboard using publicly available outputs of (sub-)national clinical or laboratory-based surveillance data was developed in 2022. Twenty-six countries from both hemispheres were included, based on the availability and consistency of surveillance data for both viruses. For each country, data were extracted on the weekly number of hMPV- or RSV-positive cases and the weekly positivity rate (cases/total tested).

**Results:**

From Jan-22­, hMPV and RSV circulated globally with seasonal patterns in most geographic regions. The hMPV season was typically observed in winter and spring, following the RSV season in autumn and winter. There was some overlap between the hMPV and RSV seasonal epidemic curves in most countries. The number of cases and the peak positivity rate were generally lower for hMPV than RSV. However, not all countries fit these patterns: hMPV can circulate year-round in tropical countries, and RSV peaked particularly early in East Asia, with a second peak in the spring and summer. While these data suggest a trend for both hMPV and RSV to return to pre-pandemic seasonality, there was residual displacement of the timing of the peaks in multiple countries.

**Conclusion:**

hMPV and RSV displayed seasonal patterns in most countries with an evolving return to pre-pandemic seasonality. Continued surveillance is required to confirm this trend during the coming season to better understand the dynamics of hMPV seasonality.

Source of funding: Funded by Icosavax, a member of the AstraZeneca Group. All authors contributed to the development of the publication and maintained control over the final content.

**Disclosures:**

Barry Rodgers-Gray, n/a, AstraZeneca: Employer has received payment from AstraZeneca for work on various projects outside the scope of this study|Sanofi: Employer has received payment from Sanofi for work on various projects outside the scope of this study John Fullarton, n/a, AstraZeneca: Employer has received payment from AstraZeneca for work on various projects outside the scope of this study|Sanofi: Employer has received payment from Sanofi for work on various projects outside the scope of this study

